# Placental Growth Factor Led Management of the Small for Gestational Age Fetus: Randomised Controlled Feasibility Study

**DOI:** 10.1111/1471-0528.70106

**Published:** 2025-12-12

**Authors:** Siân Bullough, Michelle Dower, Richard Jackson, Alexander E. P. Heazell, Kerry Woolfall, Lazaros Andronis, Louise Kenny, Zarko Alfirevic, Andrew Sharp, The PLANES study group, Emily Benbow, Gary Johnstone, Erin McClosky, Charlotte Sanders, Elizabeth Deja, Hannah Doughty, Vivan Patel

**Affiliations:** ^1^ Harris Research Centre University of Liverpool Liverpool UK; ^2^ Liverpool Women's Hospital Liverpool UK; ^3^ Department of Health Data Science in the Institute of Public Health Policy and Systems University of Liverpool Liverpool UK; ^4^ Maternal and Fetal Health Research Centre University of Manchester Manchester UK; ^5^ Department of Obstetrics, Saint Mary's Hospital Manchester University NHS Foundation Trust Manchester UK; ^6^ Department of Public Health Policy and Systems University of Liverpool Liverpool UK; ^7^ Centre for Health Economics at Warwick University of Warwick Coventry UK

**Keywords:** angiogenic, biomarker, feasibility, FGR, late growth restriction, management, PlGF, randomised, sFlt‐1, SGA

## Abstract

**Objective:**

To determine the feasibility of a trial investigating the optimal timing for the birth of women with a suspected late preterm and term SGA baby using either angiogenic biomarker‐led care or standard care.

**Design:**

A mixed methods study including a randomised feasibility trial, interviews, questionnaires and economic analysis.

**Setting:**

Two tertiary maternity hospitals in the UK.

**Population:**

Women with suspected SGA pregnancies between 32^+0^ weeks gestation and 37^+6^ weeks gestation.

**Methods:**

Women were randomised in a 3:1 ratio to biomarker‐led care versus standard care. Biomarker tests were either revealed, with birth delayed until 40 weeks if normal (sFlt‐1/PlGF < 38 pg/mL) and considered from 37 weeks if abnormal (sFlt‐1/PlGF ≥ 38 pg/mL), or concealed alongside standard care.

**Main Outcome Measures:**

Primary outcome was the feasibility of the study measured through the recruitment rate and adherence. Secondary outcomes were the qualitative, proof‐of‐concept and economic analyses.

**Results:**

Out of 128 women invited to participate 78 women were recruited giving a recruitment rate of 60.1% (95% confidence interval 52%–69%). Sixty‐seven of the 78 women consented to randomisation. Sixteen parents and 12 clinicians were interviewed. Fourty parents completed a questionnaire. Participants, partners and clinicians viewed the study as acceptable but experienced challenges in participation and delivering the study. There were no significant adverse events or differences in neonatal outcomes. Collection of health economics data was feasible.

**Conclusions:**

The clinical, qualitative and economic results support the acceptability of utilising sFlt‐1/PlGF to refine SGA management after 32^+0^ weeks but the feasibility is less certain.

## 1 Introduction

Stillbirth impacts 3.35 per 1000 births in the UK, with 25%–43% of stillbirths being small for gestational age (SGA) [1]. The risk of stillbirth at term in the UK for a fetus with a weight appropriate for its gestational age is 1 per 1000, rising to 2.3 per 1000 for SGA infants [2]. However, many SGA fetuses have no evidence of underlying placental disease and thus may be appropriately small with no excess stillbirth risk, leading to unnecessary interventions.

To reduce SGA‐related stillbirth, and in the absence of effective tests to establish fetal wellbeing, clinical management strategies moved towards early term delivery from 37 weeks' gestation [3, 4]. However, early term birth may be detrimental for some, with higher rates of behavioural problems and additional educational needs in children born prior to 40 weeks' gestation [5, 6].

Ultrasound Doppler studies are used as a marker of placental dysfunction in SGA and can effectively risk stratify foetuses prior to 32 weeks [7]. However, ultrasound Doppler is less effective after 34 weeks' gestation meaning that there is no recommended test for risk stratification of an SGA fetus towards term [8, 9, 10].

Biomarker‐led care with soluble fms‐like tyrosine kinase‐1 and placental growth factor (sFlt‐1/PlGF) ratio has allowed risk stratification and improved management of women with suspected preeclampsia [11, 12]. A Cochrane review identified sFlt‐1/PlGF as having the best potential for stillbirth prediction and there are a growing number of studies showing that the ratio can help predict adverse perinatal outcomes in SGA and FGR [13, 14, 15, 16]. Management with sFlt‐1/PlGF of reduced fetal movements in later pregnancy has been shown to be feasible [17]. Therefore, the sFlt‐1/PlGF ratio may have value in risk stratifying an SGA pregnancy, helping to reduce intervention in those who are low risk for adverse outcomes.

The aim of this study was to establish if it is feasible to perform a randomised controlled trial (RCT) of sFlt‐1/PlGF led management of the late preterm and term suspected SGA fetus. This will be assessed through the feasibility of recruiting and retaining women to the study, the acceptability of such an approach to women, partners and clinicians and health economic data.

## 2 Methods

Placental Growth Factor led Management of the Small for Gestational Age Fetus (PLANES) was a feasibility RCT conducted at two tertiary maternity hospitals in the Northwest of England. The study protocol was published prior to the study opening in Pilot and Feasibility Studies and amendments are listed in Table S1 [18].

Between April 2022 and October 2023 pregnant women with a suspected SGA fetus, between 32^+0^ and 37^+6^ weeks' gestation, were invited to participate in the study. SGA was defined as an ultrasound estimated fetal weight (EFW), less than the 10th centile on local fetal growth charts with a normal umbilical artery Doppler [19]. Growth chart standards used across the two sites were GROW and WHO [20, 21]. Multiple pregnancy, maternal age < 16 years, known or suspected structural or chromosomal fetal anomaly and severe maternal disease requiring urgent delivery were exclusion criteria.

**FIGURE 1:**
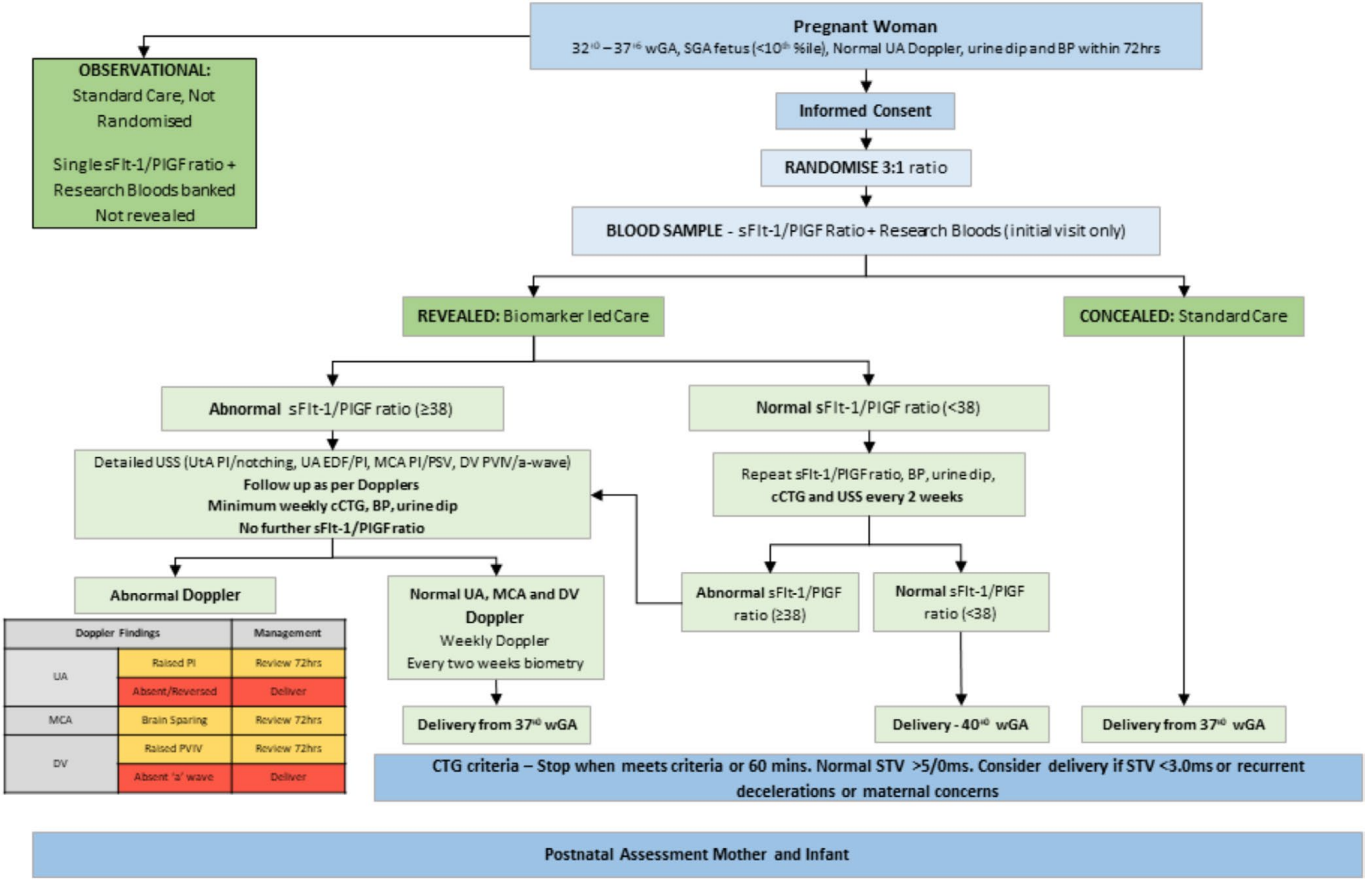
Study flow diagram.

Women were randomised to the intervention arm (revealed biomarker led care) or standard care arm (concealed biomarker with routine NHS care) in a 3 to 1 ratio, as the outcomes and opinions of those in the intervention arm were deemed of greater value to inform the study feasibility than the control arm (Figure 1). Stata v15.1 was used to create the randomisation allocation table and participants were randomised using the stratified, blocked method of allocation. Random allocation was performed using site as the sole stratification factor. Those randomised to intervention had a sFlt‐1/PlGF ratio revealed to their clinician. If normal, (< 38 pg/mL), they were offered birth at 40 weeks with repeat fetal growth scans and sFlt‐1/PlGF ratio assessment every 2 weeks. If the sFlt‐1/PlGF ratio was or became abnormal (≥ 38 pg/mL), they would have weekly Doppler assessments and repeat growth scans every 2 weeks performed by an obstetrician specialising in fetal growth and timing of birth considered from 37^+0^ weeks' gestation. Consideration for earlier timing of birth could be triggered by clinical concerns such as ultrasound concerns (e.g., abnormalities in the umbilical artery Doppler, concerns with growth velocity), fetal concerns (e.g., reduced fetal movements and abnormal antenatal CTG) or maternal concerns (e.g., severe preeclampsia and antepartum haemorrhage). Outcomes were recorded and analysed on an intention‐to‐treat basis. Participants randomised to the standard care arm had a sFlt‐1/PlGF ratio taken at recruitment, with the result concealed from the clinical and study team. No further sFlt‐1/PlGF ratio sampling was performed. Care and birth timing was in line with hospital policy and directed by a consultant obstetrician. When the study protocol was ratified in 2019, hospital policy was based on the RCOG greentop guidance advising birth from 37^+0^ weeks (hence birth from 37^+0^ stated in the flowchart in Figure 1), but once the study had opened to recruitment in 2022 hospital policy was aligned to the Saving Babies Lives Care Bundle Version 2 guidance [3, 22].

It was recognised that there was a group of women that were wishing to take part in the study but did not wish to be randomised. It was felt that there was still value in the quantitative and qualitative data from this group to inform the design of a future trial to help optimise recruitment and retention. An observational arm was included for those women. A sFlt‐1/PlGF ratio was taken and concealed and outcome data was collected. These women were managed as per the standard care arm.

### 2.1 Qualitative

Women and their birth partners were invited to complete a questionnaire and/or take part in an interview. Women and birth partners with relevant experience within the previous 3 years were also recruited via social media advertising. Staff were invited to take part in either a focus group or interview. Topic guides and the questionnaire aimed to explore women's, birth partners' and clinicians' views on the approach to feasibility RCT recruitment, covering areas such as decision making, content of study information materials, potential barriers to recruitment (Questionnaire S1, Topic Guide S1, Topic Guide S2). Respondent validation was used to add unanticipated topics to the topic guide. Based on previous trial feasibility studies, it was anticipated that we would need to interview 15–25 parents, conduct 1–2 focus groups and receive approximately 40 questionnaires (~50% of the target sample including those who declined to participate) to reach information power [23, 24, 25, 26]. Thematic reflexive analysis of qualitative interview and focus group data was broadly interpretive and inductive which refers to a systematic process where the researchers aimed to collect and analyse data without preconceived ideas or theories about what they would find [27]. Nvivo 12 software was used to assist the organisation and coding of data [28]. Questionnaire data were analysed using descriptive statistics.

### 2.2 Economic

The feasibility of collecting data required for conducting an economic evaluation in a future RCT was assessed. Two key categories of economic data were of interest: (i) inputs, in the form of use of health care resources (e.g., care related to birth and complications) and (ii) outcomes (maternal health‐related quality of life (HRQoL) and childbirth experience). HRQoL was measured through the EuroQol EQ‐5D (5‐level version) and childbirth experience was assessed through the Childbirth Experience Questionnaire (CEQ) [29, 30]. Data on maternal HRQoL were requested from all PLANES participants and were collected at two points: at enrolment (T1) and shortly after delivery (T2). CEQ was collected at time point T2 only. Data on use of health care resources were collected through the PLANES case report form, drawing on data from participants' medical records.

### 2.3 Statistical Analysis

Continuous data were summarised as median (IQR) and categorical data are summarised as frequencies of counts with associated percentages. Confidence intervals (CI) about percentages are reported assuming binomial proportions. Outcomes and analyses are predominantly exploratory in nature. Tests between groups are provided but are not attached to any pre‐determined study hypothesis. Comparisons of data between groups are performed using Fisher's exact test or Wilcoxon's signed‐rank test as appropriate. A *p*‐value of 0.05 was considered statistically significant. All analyses were performed using R (V4.2.1).

### 2.4 Outcomes

The primary outcome was study feasibility and this was assessed through the ability to recruit women, compliance with the study management pathways and women's experiences of the study. Feasibility outcomes were defined as the number of women recruited and randomised and the number of women/clinicians whose management was compliant with the study protocol. The recruitment target was set at 100 women, 50 from each site. Secondary outcomes included fetal outcomes (stillbirth, neonatal death, Apgar < 7 at 5 min, umbilical artery pH < 7.05, birthweight < 10th centile, admission to neonatal unit and length of stay, therapeutic cooling, length of stay in hospital, duration of respiratory support) and maternal outcomes (gestation at birth, frequency of induction of labour or planned caesarean section, maternal hypertensive disorders, intensive care admission and maternal death) that were collected to assess proof of concept but the study was not powered to examine differences in these outcomes.

Trial data are presented in accordance with the CONSORT guidelines. The CONSORT checklist can be found in Table S2. The published study protocol contains details on trial governance procedures and adverse event reporting [18]. The data management committee regularly reviewed the progress of the study and there were no concerns raised from adverse outcomes and there were no serious adverse outcomes during the study.

## 3 Results

### 3.1 Feasibility Outcomes

**FIGURE 2:**
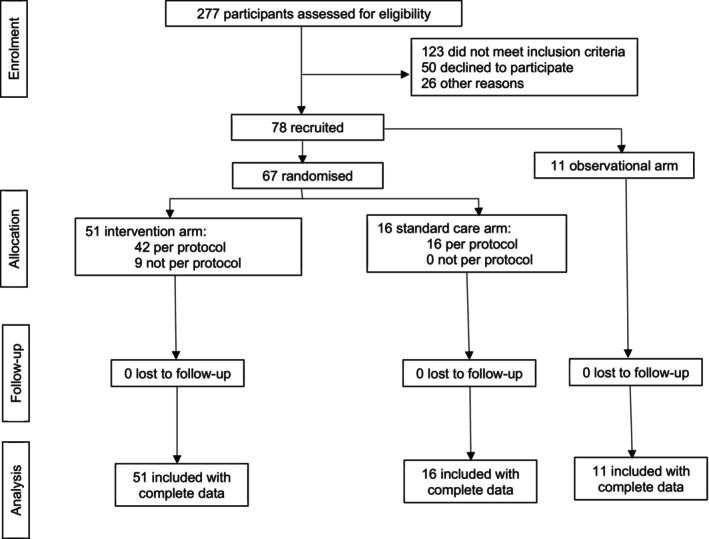
Consort diagram for PLANES study.

The PLANES study opened in April 2022 at the lead site and February 2023 at the second site and closed to recruitment in November 2023. Two hundred and seventy‐seven women were screened to be eligible for the study (Figure 2). Of those who met the inclusion criteria (*n* = 128) 50 declined and 78 consented, giving a 60.1% (95% CI 52%–69%) recruitment rate. 67 (85.9%, 95% CI 78%–94%) consented to randomisation and 11 (14.1%, 95% CI 6%–22%) declined randomisation (Table 1). Four out of the 11 women who declined randomisation completed questionnaires but none of them shared their views on why. Seven out of 11 women declining randomisation had a planned caesarean birth prior to recruitment. All 50 women and their birth partners who declined to participate in the study also declined participation in the qualitative assessments.

**TABLE 1:** Primary outcomes and maternal characteristics and fetal parameters at recruitment.

Women assessed for eligibility	277 women
Eligible women	128 (46%, 95% CI 40–52) women
Participants recruited	78 (60%, 95% CI 52–69) women
Participants randomised	67 (86%, 95% CI 78–94) women

	Intervention	Standard care	Observational
	(*n* = 51)	(*n* = 16)	(*n* = 11)
Age (years): median (IQR)	30 (26–34)	33 (28.3–38)	28 (25.5–29)
BMI: median (IQR)	27.0 (23.9–34.2)	27.4 (22.8–32.1)	25.0 (24.1–33.5)
Ethnicity			
White	42 (82%)	12 (75%)	9 (82%)
Asian	1 (2%)	1 (6%)	1 (9%)
Black African/Caribbean	3 (6%)	0 (0%)	1 (9%)
Other	5 (10%)	3 (19%)	0 (0%)
Smoking status			
Current smoker	6 (12%)	5 (31%)	3 (27%)
Never smoked	31 (61%)	10 (62%)	8 (73%)
Ex smoker	4 (27%)	1 (6%)	0 (0%)
Nulliparous	18 (35%)	2 (12%)	3 (27%)
Gestation (weeks): median (IQR)	34^+1^ (33^+1^–34^+6^)	33^+4^ (32^+4^–34^+6^)	34^+5^ (33^+6^–35^+5^)
Estimated Fetal Weight (grams): median (IQR)	1898.0 (1688.5–2064.0)	1862.0 (1722.25–2035.25)	2007.0 (1645.5–2227.5)
Estimated fetal weight (EFW) centile—GROW: median (IQR)	4.0 (2.3–7.8)	6.4 (5.2–8.6)	3.2 (1.1–4.0)
EFW 3rd–< 10th Centile—GROW	25 (49%)	10 (62%)	5 (45%)
EFW < 3rd Centile—GROW	17 (33%)	3 (19%)	5 (45%)
Umbilical artery pulsatility index: mean (standard deviation)	0.98 (0.84–1.07)	0.92 (0.81–1.03)	0.90 (0.77–1.01)
Pre‐eclampsia at recruitment	1 (2%)	0 (0%)	0 (0%)
Systolic blood pressure: Median (IQR)	119.0 (113.8–126.0)	115.5 (106.6–118.3)	108.5 (106.5–115.5)
Diastolic blood pressure: Median (IQR)	69.0 (64.0–73.0)	60.5 (57.9–68.8)	65.0 (61.5–70.5)

*Note:* All results presented as *n* (percentage) unless otherwise stated. Primary feasibility outcomes have 95% confidence intervals (CI) provided around the percentage result.

Abbreviations: BMI, body mass index; GROW, gestation‐related optimal weight chart; IQR, interquartile range.

82.4% (95% CI 72%–93%) of women in the intervention arm had management compliant with the protocol. Of the eligible women 7 (17.5%, 95% CI 6%–29%) gave birth from 40^+0^ weeks' gestation. 72.7% (95% CI 58%–88%) of women who gave birth prior to 40^+0^ weeks' gestational age had other medical indications for earlier birth, in keeping with the protocol. Table S3 details the indications for birth less than 40^+0^ weeks' gestational age in those with normal sFlt‐1/PlGF values.

Of the nine women who were considered to have not received the allocated intervention, five were due to the woman's request for earlier birth and four were due to clinician decision to deviate from the protocol. Five of these women had a planned caesarean birth date already arranged prior to enrolment into the study.

Eleven women had abnormal sFlt‐1/PlGF results in the intervention arm. The indications for birth were, five women for maternal condition (preeclampsia (*n* = 4) and antepartum haemorrhage (*n* = 1)), three for abnormal umbilical artery Dopplers, one for abnormal antenatal CTG and two for abnormal biomarker result alone (one at 37^+2^ and one at 39^+0^ weeks' gestational age).

### 3.2 Women, Birth Partner and Clinician Perspectives

Sixteen women and birth partners (12 mothers and 4 fathers) participated in an online interview (Figure S1). A total of 40 parents (37 mothers, 3 fathers) completed a questionnaire. Twelve clinicians participated in the focus groups and interviews including six midwives, four doctors and two advanced clinical practitioners (Table 2).

**TABLE 2:** Parent questionnaire responses (*n* = 40).

Statement	Response, *n* (%)		
	Agree	Neither agree nor disagree	Disagree
a. The doctor or nurse checked that it was a convenient time to discuss research before discussing the PLANES study	40 (100)	0 (0)	0 (0)
b. The information I received about the study was clear and straightforward to understand	40 (100)	0 (0)	0 (0)
c. I had enough opportunity to ask questions	40 (100)	0 (0)	0 (0)
d. I was satisfied with the consent process^a^	39 (100)	0 (0)	0 (0)
e. It was difficult to take in the information I was given about the study^a^	3 (8)	4 (11)	31 (82)
f. It was difficult to make a decision about participating in the study	6 (15)	7 (18)	27 (68)
g. I made the decision^a^	36 (92)	3 (8)	0 (0)

^a^ Missing responses: Statement (d) *n* = 1, (e) *n* = 2 and (g) *n* = 1.

**FIGURE 3:**
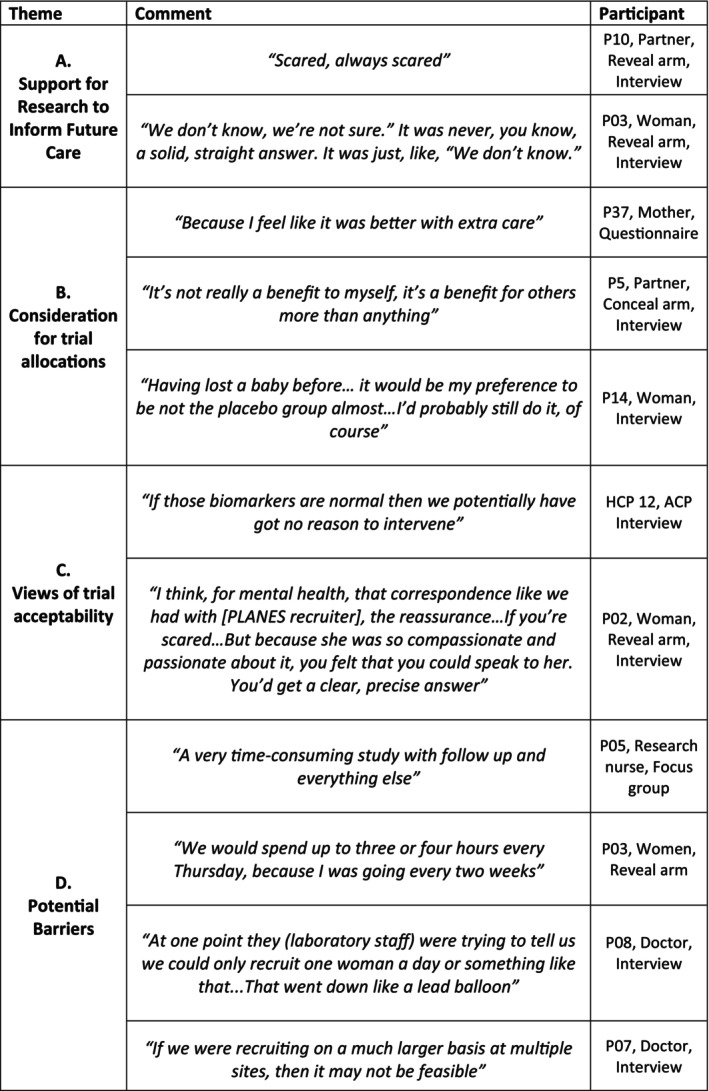
Patient, partner and health care professional views and comments from interviews grouped into themes.

Interviews revealed the emotional burden of an SGA diagnosis. Many described the fear, uncertainty and upset they felt when they were told their baby was SGA. Some were frustrated that clinicians could not provide them with a clear answer about what the diagnosis would mean for the management and subsequent outcomes for their baby. Uncertainty about clinical management appeared to influence views on the proposed PLANES trial. Across interviews, focus groups and questionnaires women, birth partners and clinicians stated their support for research to inform the future care of SGA pregnancies (Figure 3, section A).

Women and birth partner questionnaire responses are shown in Table 2. Three women stated that they declined participation in PLANES although they did not elaborate on a reason.

Many described their preference for being allocated to the intervention arm due to the belief they would receive extra care. Nevertheless, when asked to reflect on how they would feel if they had been allocated to the standard care arm, all stated they would still participate. Indeed, those allocated to the standard care arm were positive about the study and wanted to take part to hopefully benefit others in the future (Figure 3, section B). Some suggested that “*some reassurance*” (P15, Woman, SM interview) would be needed for parents randomised to the standard care arm to clarify they would receive all the support they would need.

The trial was viewed as acceptable as many felt biomarker‐led care may help provide additional reassurance whilst potentially reducing unnecessary interventions. Some randomised parents felt they benefitted from additional information and emotional support (Figure 3, section C).

Parents reported barriers to participation including the burden of lengthy regular appointments involving blood tests and associated costs for parking. Staff also described challenges in their capacity to deliver the study and some questioned the feasibility of conducting a larger study (Figure 3, section D).

### 3.3 Proof of Concept Analysis

Maternal characteristics and fetal parameters at recruitment based on study arm are displayed in Table 1. The median gestational age of birth was 38^+4^ (IQR 37^+1^–39^+1^) in the intervention arm compared to 38^+0^ (IQR 37^+3^–38^+5^) in the standard care arm (*p* = 0.85). In the intervention arm, 5 women (10%, 95% CI 2%–18%) went into spontaneous labour, 31 (61%, 95% CI 47%–74%) underwent induction of labour and 15 (29%, 95% CI 17%–42%) had planned pre‐labour caesarean births. In the standard care arm, 2 (12.5%, 95% CI 0%–29%) went into spontaneous labour (1 case of preterm labour at 34^+4^ weeks gestational age), 10 (62.5%, 95% CI 39%–86%) underwent induction of labour and 4 (25%, 95% CI 4%–46%) had planned pre‐labour caesarean births. 28 (55%, 95% CI 41%–69%) women in the intervention arm had a vaginal birth compared to 9 (56%, 95% CI 32%–8%) in the standard care arm and 8 (16%, 95% CI 6%–26%) had an emergency caesarean birth in the intervention arm compared to 3 (19%, 95% CI 0%–38%) in the standard care arm. 4 (8%, 95% CI 0%–15%) women developed preeclampsia in the intervention arm compared to none in the standard care arm (*p* = 0.83). There were no serious adverse maternal outcomes.

The median birthweight of babies born in the intervention arm was 2655 g (IQR 2354–2850) compared to 2550 g (IQR 2398–2903) in the standard care arm (*p* = 0.96). The median birthweight centiles according to GROW were 4.3rd centile (1.7–11.4) in the intervention arm and 4.4th centile (3.2–5.6) in the standard care arm (*p* = 0.11) [20]. 15 (29%, 95% CI 17%–42%) were SGA (between 3rd centile and < 10th centile) and 20 (39%, 95% CI 26%–53%) were FGR (< 3rd centile) in the intervention arm. 3 (19%, 95% CI 0%–38%) were SGA and 7 (44%, 95% CI 19%–68%) were FGR in the standard care arm. This gives a false positive rate of 32% and 37% in the intervention and standard care arms, respectively. Five (10%, 95% CI 2%–18%) babies in the intervention arm were admitted to the neonatal unit compared to 1 (6%, 95% CI 0–18) in the standard care arm. No babies required therapeutic cooling or respiratory support and there were no other adverse neonatal outcomes.

### 3.4 Post Hoc, Secondary Analysis

Table 3 displays the maternal and neonatal outcomes based on study arm and management pathway. There were no abnormal sFlt‐1/PlGF results in those randomised to the standard care arm but repeat sampling was not performed in this arm. Of the 12 women that had abnormal sFlt‐1/PlGF results (11 in the intervention arm and one in the observational arm), eight were abnormal at recruitment and four became abnormal as part of routine re‐testing every 2 weeks in the intervention arm.

**TABLE 3:** Maternal and neonatal outcomes analysed depending on randomisation and sFlt‐1/PlGF pathway in the intervention arm.

	Intervention		Standard care	*p*	
	*N* = 51				
	Normal	Abnormal		Intervention vs. Standard Care	Intervention Normal vs. Standard Care
	sFlt‐1/PlGF	sFlt‐1/PlGF	*N* = 16		
	*N* = 40	*N* = 11			
Gestation at delivery: Median (IQR)	38^+1^ (37^+4^–39^+3^)	37^+0^ (35^+6^–37^+6^)	38^+0^ (37^+3^–38^+5^)	0.850	0.910
Onset of labour					
Spontaneous	5 (12%, 2–23)	0 (0%, 0–17)	2 (12%, 0–29)	0.823	1.00
Induction	26 (65%, 50–80)	5 (45%, 16–75)	10 (63%, 39–86)		
Planned caesarean	9 (22%, 10–35)	6 (55%, 25–84)	4 (25%, 4–46)		
Mode of delivery					
Vaginal birth	26 (65%, 50–80)	2 (18%, 0–41)	9 (56%, 32–81)	1.00	0.668
Caesarean					
Emergency	5 (12%, 2–23)	3 (27%, 1–54)	3 (19%, 0–38)		
Planned	9 (22%, 10–35)	6 (55%, 25–84)	4 (25%, 4–46)		
Pre‐eclampsia	0 (0%, 0–5)	4 (36%, 8–65)	0 (0%, 0–12)	0.830	NA
Apgar: median (IQR)	10 (9–10)	10 (9.5–10)	10 (10–10)	0.192	0.267
Umbilical artery pH: median (IQR)	7.22 (7.18–7.28)	7.27 (7.21–7.33)	7.26 (7.21–7.30)	0.801	0.635
Birthweight (grams): median (IQR)	2680 (2535–2875)	2150 (1930–2495)	2550 (2398–2903)	0.956	0.382
Birthweight centile – GROW: median (IQR)	6.1 (2.2–12.6)	1.8 (0.4–7.7)	4.4 (3.2–5.6)	0.109	0.275
3rd – < 10th centile – GROW	12 (30%, 16–44)	3 (27%, 1–54)	3 (19%, 0–38)	0.036	0.035
< 3rd centile – GROW	14 (35%, 20–50)	6 (55%, 25–84)	7 (44%, 19–68)	0.516	0.731
Admission to SCBU	3 (8%, 0–16)	2 (18%, 0–41)	1 (6%, 0–18)	1.00	1.00
Length of stay on SCBU: median (IQR)	8 (4.5–29.0)	18.5 (14.3–22.8)	10 (10–10)	—	—

*Note:* All results presented as n (percentage, 95% confidence interval (%)) unless otherwise stated.

Abbreviations: GROW, gestation related optimal weight chart; IQR, interquartile range; SCBU, special care baby unit.

**FIGURE 4:**
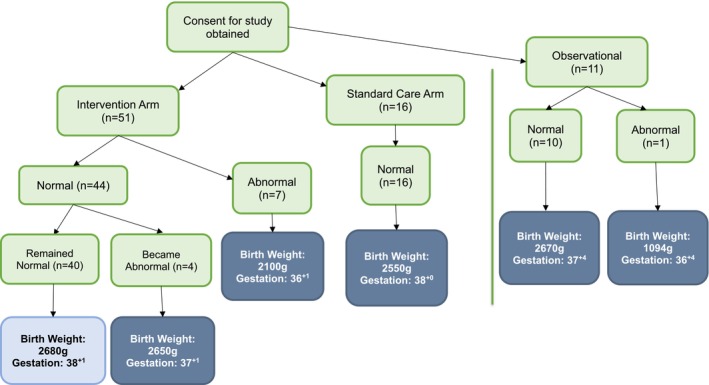
Displays a Wilcoxon test analysis comparing gestational age at delivery for those in the intervention arm with a normal sFlt‐1/PlGF result against all other groups (*p* = 0.012). The mean gestational age at birth for those in the intervention arm with a normal biomarker was 38^+1^ weeks (standard deviation (SD) = 13.1). This was compared to 37^+1^ (SD = 1) in the intervention arm with a biomarker result that became abnormal, 36^+1^ (SD = 3.2) in the intervention arm with an abnormal biomarker result at entry to the study, 38^+0^ (SD = 1.3) in the standard care arm, 37^+4^ (SD = 0.8) in the observational arm with a normal biomarker result and 36^+4^ (SD NA) in the observational arm with an abnormal biomarker result.

The randomised standard care arm was combined with the observational arm to create a combined standard care arm. The median gestational age of delivery was 38^+4^ (IQR 37^+1^–39^+1^) in the intervention arm compared to 37^+4^ (IQR 37^+3^–38^+3^) in the combined standard care arm (*p* = 0.95). Figure 4 displays a Wilcoxon test analysis comparing gestational age at birth for those in the intervention arm with a normal sFlt‐1/PlGF result against all other groups. This suggests that those in the intervention arm with a normal sFlt‐1/PlGF result gave birth at a later gestation (38^+1^ weeks) compared to those in all the other groups.

### 3.5 Economic Analysis

Forty‐nine (63%) participants provided completed EQ‐5D‐5L data. Of the 29 participants who did not return complete EQ‐5D‐5L data, 19 (66%) returned neither of the questionnaires at T1 and T2, and 10 (35%) failed to return the questionnaire at T1. Completion of CEQ was the same. Participants with complete and incomplete EQ‐5D‐5L data were broadly similar in relation to key characteristics (Table S4A). Illustrative analyses were carried out to identify possible trends in HRQoL and these are displayed in Table S4B–D.

Overall, the feasibility of collecting economic data for the comparison of interest suggests fair return rates for patient‐completed HRQoL and availability of complete and detailed data around key NHS care use.

## 4 Discussion

### 4.1 Main Findings

Overall, the feasibility of such an approach is not fully supported due to difficulties with recruitment and compliance. The quantitative, qualitative and economic analyses support the acceptability of the PLANES study and have helped identify areas that would need to be adapted to support a successful future large‐scale RCT of biomarker‐led management of SGA pregnancies. Qualitative analysis highlighted the burden of SGA pregnancies to families and the desire for more support and improved care for these women.

This study was not powered to assess clinical outcomes. There are no differences observed between the randomised groups. Proof of concept may be supported by a trend towards birth at a later gestation in the intervention arm in women with a normal sFlt‐1/PlGF result in the post hoc analysis.

### 4.2 Strengths and Limitations

The PLANES study is one of the first studies to assess the potential role that sFlt‐1/PlGF testing could play in risk stratifying late preterm and term SGA pregnancies in a UK healthcare setting [31]. It's the only study to have used a mixed‐methods approach to also address possible qualitative and economic components which would be necessary to inform any practice change within the National Health Service (NHS).

The study did not recruit to its predefined target and factors influencing this will be discussed. As the nature of the study was feasibility this does not impact the validity of the results presented and it allows insights into how a future RCT design could be delivered. The differences in recruitment between the two sites, and results from the qualitative analysis, highlight likely challenges a larger RCT would face and could limit the external validity. There may be a need to simplify the study pathway to allow it to fit more easily alongside current clinical practice within the NHS.

We had limited insight from those women and birth partners who declined to participate in any aspect of the study. This would have been valuable information to inform any future study design. Of additional value would have been the views of women and their birth partners on outcomes of importance for a future trial but this was not explored in this study.

In relation to the use of health care resources, key procedures relate to examinations and care provided within NHS hospitals, where the majority of costs relevant to the comparison accrue. A larger, subsequent study would allow collecting data on NHS resource use outside hospitals and private expenditures (out‐of‐pocket payments) incurred by participants. However, due to the nature of the intervention, it is expected that little, if any care, relevant to the participant's fetal status is received outside hospitals/clinics, so the participant burden of providing such information is expected to be minimal.

### 4.3 Interpretation

Despite our predefined feasibility criteria being largely met, with a recruitment rate of 60.1% (95% CI 52%–69%), an 85.9% (95% CI 78%–94%) randomisation rate and a compliance rate of 82.4% (95% CI 72%–93%), there were still challenges and the prespecified recruitment target of 100 women was not met. These challenges were echoed in the qualitative findings. The Covid‐19 pandemic resulted in significant delays in the opening of the second site and likely played a role in slow initial recruitment, but this also highlights the challenges of recruiting to such a study beyond its lead site. Not all women will want the opportunity to extend their pregnancy, such as due to preplanned caesarean birth, and this could impact a true randomised population in any potential future study. Other challenges included women being offered dates for early term births prior to entry to the study and a proportion of women not recruited for ‘other’ reasons, 38.5% (10 out of 26), because clinicians deemed it inappropriate despite meeting the inclusion criteria. This may reflect that many healthcare providers still do not feel confident offering later birth in the context of SGA after many years of birth being advised from 37^+0^ weeks in the UK. This is despite more recent guidance bringing UK practice more in keeping with similar countries [4, 8, 32, 33]. These issues have the potential to be alleviated as time may improve compliance with the new RCOG guidance advising later birth, between 39^+0^ and 39^+6^, but it warrants careful consideration for future study planning with regards to simplification of the protocol and the outcomes to be assessed [8, 32]. Patient interviews also identified a desire for some to be in the intervention arm. It will be important to consider patient preferences and clinician biases to inform the development of any future study design to optimise recruitment and retention. The economic analysis commented that a subsequent trial will need to consider ways of increasing the return rates of the HRQoL, which may be through further adjustments in the timing and practicality of completing HRQoL questionnaires.

During the study there has been significant change in national guidance with regard to the timing of birth for pregnancies complicated by SGA [8, 32]. The conception of the PLANES study, 10 years ago, was due to guidance advising birth at 37 weeks for an SGA fetus [3]. In that time national guidance has gradually moved, through 3 iterations of the Saving Babies Lives Care Bundles, from 37^+0^ weeks to now advising birth by 39^+6^ weeks gestational age [22, 32]. Some may therefore feel that there is no incremental value of such a study design as national UK guidance has largely come into line with that of the intervention arm of the study with birth being delayed, for SGA, until 39^+6^ weeks gestational age [8, 32]. Nevertheless, we propose that greater evidence of fetal wellbeing, or accuracy of diagnosis of FGR or SGA, is needed. A recent RCT demonstrated that sFlt‐1/PlGF can perform as well as conventional Doppler assessment to decide the timing of birth in FGR and SGA and may improve neonatal and maternal outcomes [31]. In line with our results, they also demonstrated a prolongation of gestation at birth in those managed with sFlt‐1/PlGF (39.0 weeks, IQR 37.9–40.0) compared to routine care with Doppler (38.4 weeks, IQR 37.6–39.7) [31]. Due to the absence of qualitative and economic methodologies, to assess acceptability to participants and healthcare providers and consider financial implications, in the aforementioned RCT we feel it is unlikely sFlt‐1/PlGF will replace Doppler examination. We eagerly await the outcome of the TRUFFLE 2 RCT which may bring clarity to the use of UCR Doppler to guide the timing of birth for late FGR and provide additional evidence on how sFlt‐1/PlGF may improve diagnostic accuracy [34, 35]. We believe sFlt‐1/PlGF could form an important part of late preterm and term SGA management, with the potential to improve fetal and maternal health whilst offering more maternal choice, but more clarity is needed on its prognostic accuracy for adverse outcomes in later pregnancy and its capability when combined with newer Doppler parameters. The POPS 2 study will go some way in addressing these questions [36].

A further study would require careful consideration of the most appropriate primary outcome, as significant adverse events such as stillbirth and neonatal death are rare outcomes, even in high‐risk cohorts [2, 37]. To power for such outcomes would require a sample size of over 30 000 women with SGA. This would be too challenging and continues to be a research challenge for studies of SGA at later gestations [17]. Prolongation of gestation, supported by our post hoc analysis and Garcia‐Manau et al., and composite neonatal outcomes may be a more appropriate measure, alongside maternal qualitative data about the impact on birth choices and experiences and the impact on service provision, such as for induction of labour [31, 35, 38, 39].

## 5 Conclusion

The clinical, qualitative and economic results of our study support the acceptability of utilising sFlt‐1/PlGF to guide SGA management after 32^+0^ weeks' gestation but the feasibility of such an approach is less certain due to difficulties with recruitment and compliance.

Recent changes in clinical practice within the UK, to delay birth in SGA pregnancies until 39–40 weeks gestational age, but without additional assessments of fetal wellbeing, mean the future scope of sFlt‐1/PlGF ratio informed management of SGA from 32^+0^ weeks is more challenging [8, 32]. This means that careful consideration of study design, such as streamlining interventions and more prescriptive delivery timing endpoints, and a collaborative research group approach is essential. Despite the challenges, women, their partners and clinicians, affected by and involved in SGA management, have shown that this work is important and needed. We propose that greater evidence of fetal wellbeing, in the context of SGA at term, is needed. sFlt‐1/PlGF could form an important part of this, but more data are needed on its prognostic ability at later gestations and how this would complement current clinical practice.

## Author Contributions

S.B. wrote the manuscript and analysed data, M.D. performed data acquisition, R.J. provided statistical review, A.E.P.H. critically reviewed the manuscript, K.W. performed the qualitative analysis, L.A. performed economic analysis, L.K. critically reviewed the manuscript, Z.A. critically reviewed the manuscript, A.S. conceived the project, sought funding and oversaw the project and manuscript throughout. (E.B., G.J., E.M.C., C.S., H.D., E.D., V.P. all assisted with data collection). All authors reviewed the final manuscript.

## Funding

This project is funded by the National Institute for Health and Care Research (NIHR) under its Research for Patient Benefit (RfPB) Programme (Grant Reference Number PB‐PG‐0817‐20021). The views expressed are those of the author(s) and not necessarily those of the NIHR or the Department of Health and Social Care.

## Ethics Statement

The study received a favourable ethical opinion from the North East Research Ethics Committee, REC reference 18/NW/0857, and the study was registered with ISRCTN, number 58254381 http://www.isrctn.com/ISRCTN58254381.

## Conflicts of Interest

The authors declare no conflicts of interest.

## Supporting information


**Figure S1:** Parents and birth partner interview and questionnaire participant flow chart.

**Data S1:** Questionnaire S1: Women and partner questionnaire.

**Data S2:** Topic Guide S1: Parent interview topic guide.

**Data S3:** Topic Guide S2: Clinician interview and focus group topic guide.

**Table S1:** Protocol amendments after the publication of the protocol.

**Table S2:** CONSORT checklist.

**Table S3:** Reasons for timing of birth outside of protocol in those in the biomarker led, normal arm of the study.

**Table S4:** Health economics results. (A) Demographic characteristics of participants with complete and incomplete EQ‐5D data. (B) EQ‐5D completeness by randomisation arm. (C) Frequencies of EQ5D profiles at baseline and follow‐up. At time T1 (before delivery), the most commonly reported health state was that of ‘no problems’ in any of the EQ‐5D dimensions (11111), whilst in T2 (after delivery), the most commonly reported state was of some pain/discomfort, but no problems in other dimensions (11121). (D) Summary statistics for EQ‐5D index (utility) scores at times T1 (Enrolment) and T2 (Post‐delivery). EQ‐5D profiles were converted into index values (utility values) using the value set currently recommended by the National Institute for Health and Care Excellence in the UK^1^. On average, participant in the intervention arm had a higher EQ‐5D index (utility) scores than their counterparts in the standard care arm and observation arm. However, whilst utility scores in the intervention arm decreased markedly between T1 (enrollment) and T2 (post delivery), utility scores for the other arms remain at the same level or increased modestly. ^1^NICE. Position Statement on Use of the EQ‐5D‐5L Value Set for England.

## Data Availability

The data that support the findings of this study are available from the corresponding author upon reasonable request.
