# Supplementary material for: Placental Growth Factor Led Management of the Small for Gestational Age Fetus: Randomised Controlled Feasibility Study
Source: BJOG. 2025 Dec 12;133(4):626–37. doi: 10.1111/1471-0528.70106 (PMC12884213; doi:10.1111/1471-0528.70106)
Supplement: Supplementary file 1 — Figure S1: Parents and birth partner interview and questionnaire participant flow chart. [file BJO-133-626-s006.docx]

**PLANES embedded study parent recruitment**

**Interviews**

16 Interviewed: 12 PLANES trial, 4 social media

12 Mothers: 8 PLANES trial 4 social media)

4 Fathers: 4 PLANES partners, 0 social media)

(9 reveal arm, 3 conceal arm)

**Questionnaires**

40 returned questionnaires: 34/40 site 1, 6/40 site 2

37 mothers, 3 fathers

4 observational arm

26 reveal arm, 10 conceal arm

Recruited via social media

PLANES (78 participants, 72 site 1, 6 site 2)

11/78, 14% declined randomisation

57/78, 73% consented to questionnaire

41/78, 53 % mothers and 12 partners (total 53 parents) consented to contact for interview

6/11, 55% observational arm (5 consented to questionnaire, 1 to interview)

10 registered interest in an interview

10/10 were eligible

6/10 No response to contact

**Interviews**

10/53 No response to contact

3/53 withdrew from interview contact

4/53 No response at arranged interview

36/53 Not contacted due to saturation

7/8 women who had an abnormal sFlt-1/PIGF ratio result completed a questionnaire
