# Supplementary material for: Placental Growth Factor Led Management of the Small for Gestational Age Fetus: Randomised Controlled Feasibility Study
Source: BJOG. 2025 Dec 12;133(4):626–37. doi: 10.1111/1471-0528.70106 (PMC12884213; doi:10.1111/1471-0528.70106)
Supplement: Supplementary file 2 — Data S1: Questionnaire S1: Women and partner questionnaire. [file BJO-133-626-s008.docx]

**PLANES Placental Growth Factor Led Management of the Small for Gestational Age Fetus: A Feasibility Study.**

|  |  |  |  |  |  |  |  |  |  |  |
| --- | --- | --- | --- | --- | --- | --- | --- | --- | --- | --- |

**Participant / Partner Identification Number**:

**The following questions are about the PLANES study that you / your partner were invited to take part in. We would appreciate your views on how you were invited to participate in the study, including whether you think it is acceptable to run a larger PLANES trial across the UK.**

**We refer to people agreeing to take part in research as ‘consenting’.**

**Please Complete:**

|  |  |  |  |  |  |  |  |  |  |
| --- | --- | --- | --- | --- | --- | --- | --- | --- | --- |

**Today’s date:**

**Are you: Male** **Female**

**Participant (Mother)** **Partner (Father)** **Partner Other ______________________**

*(Please specify*)

1. Please indicate how strongly you agree or disagree with the following statements by placing a circle around the answer that best fits your opinion or decision.

| **Statements** | **Agree** | **Neither agree  nor disagree** | **Disagree** |
| --- | --- | --- | --- |
| 1. The doctor or nurse checked that it was a convenient time to discuss research before discussing the PLANES study | 1 | 2 | 3 |
| 1. The information I received about the study was clear and straightforward to understand | 1 | 2 | 3 |
| 1. I had enough opportunity to ask questions | 1 | 2 | 3 |
| 1. I was satisfied with the consent process | 1 | 2 | 3 |
| 1. It was difficult to take in the information I was given about the study | 1 | 2 | 3 |
| 1. It was difficult to make a decision about participating in the study | 1 | 2 | 3 |
| 1. I made the decision | 1 | 2 | 3 |
| 1. It was a joint decision (e.g. between patient and birth partner) | 1 | 2 | 3 |
| 1. Someone took the decision away from me | 1 | 2 | 3 |
| 1. I was not in control of the decision | 1 | 2 | 3 |
| 1. The decision was inappropriately influenced by others   If the answer to this statement is ‘Agree’,  please state who you think influenced the decision about the research: | 1 | 2 | 3 |

1. Did you / your partner consent to take part in the PLANES study?

 Yes (Go to question 3) No (Go to question 4)

1. What were your / your partner’s reasons for providing consent?

Please tick all that apply and then circle your main reason (e.g.
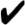
 )

| 1. To help my baby |  |
| --- | --- |
| 1. To help inform the care of babies in the future |  |
| 1. I felt that medical studies like PLANES are important |  |
| 1. Because I trusted the doctor or midwife who explained the study |  |
| 1. I didn’t feel comfortable saying no to the doctor or midwife who explained the study |  |
| 1. Other (Please state): |  |

1. If you did not provide consent, please give your reason(s) below.
2. Do you think it is acceptable to conduct a larger PLANES trial in hospitals across the UK?

 Yes No

Please explain your reasons why you think it is acceptable OR not acceptable to conduct a larger PLANES trial:

Please provide any comments or suggestions you have to improve the recruitment and consent process for the PLANES study:

**Thank you for completing this questionnaire**

**Please place your questionnaire in the envelope provided and return it to the doctor or midwife**

*Original sent to the Centre for Women’s Health Research, 1 Copy retained at site*
