# Supplementary material for: Placental Growth Factor Led Management of the Small for Gestational Age Fetus: Randomised Controlled Feasibility Study
Source: BJOG. 2025 Dec 12;133(4):626–37. doi: 10.1111/1471-0528.70106 (PMC12884213; doi:10.1111/1471-0528.70106)
Supplement: Supplementary file 3 — Data S2: Topic Guide S1: Parent interview topic guide. [file BJO-133-626-s002.docx]

**Researcher Introduction and Gaining Consent**

Can I just check that you have all had a chance to read the information sheet and complete the consent form? (If not, please could you complete it now?) Check for any questions

My name is Erin and I am a researcher at the University of Liverpool and part of the PLANES qualitative research team led by Dr Kerry Woolfall. In addition to talking with you I have been interviewing parents who have participated in the study to learn more about their experiences.

The purpose of this interview is to explore your views on the PLANES study. I will ask some questions on your current practice, proposed trial arms and potential design of a future study.

With your consent, I will audio record this session. Your views will remain confidential. The recording will be transcribed by a professional transcription company and anonymised with all names and participating hospital details removed. Only the PLANES qualitative study team – that’s myself and Kerry will see this in full.

Are there any questions before we begin?

***Check consent for audio recording – press record***

**Section 1: Role and Background of Participants**

- Please tell me what your role is at this hospital.
- Are you involved in the clinical care of children?
- How much, if any, experience do you have in recruiting to clinical trials?
- Could you please tell me how you have been involved in PLANES (e.g. your role, such as screening or recruitment)

**Section 2: Current practice and management of SGA pregnancies**

- Could you please describe to me what your usual practice is for the management of SGA?
- Did you/your unit ever use biomarker led management for SGA prior to involvement in the PLANES study? How does implementing the biomarker blood test enhance care for SGA pregnancies?

**Section 3: Screening and recruiting in the PLANES Feasibility Study**

**If screening:**

Were you involved in screening for PLANES? If yes:

- Could you talk me through the study screening process?
- How many women have you screened so far for SGA at the maternity assessment unit?
- Have any potential participants been missed? If so, please could you tell me a bit more about this
- Do you think the screening process could be improved if we moved to a full trial comparing intervention versus expectant management of placental small gestational growth?

**Recruitment:**

Were you involved in recruitment conversations with parents for PLANES? If Yes:

- *Could you tell me how you explain the PLANES feasibility study to parents? Prompt:*  Do you present each of the intervention arms (biomarker led or standard care)?
- At what time have you usually approached parents? (Explore minimum and maximum time frames)
- Thinking about your experience of recruitment in the PLANES feasibility study, how do you gauge when is best to approach parents about the study? *Explore: do you speak to others on the clinical care team? Have you ever asked the clinical team to introduce you to the parents?*
  - How do you present the benefits and risks of taking part in the study? *identify whether all staff are presenting the risks in the same way/see what phrases they use)*
  - How long have mothers / birth partners taken to consider study participation? *Explore: if there have been any issues in obtaining a consent decision from mothers / birth partners*
  - Have you encountered any difficulties in approaching the study with mothers / birth partners about taking part in research?
  - In general, how have mothers / birth partners responded to the PLANES feasibility study? (explore if any differences between those allocated to the revealed or concealed arm)
- Could you tell me if there is anything that mothers and partners tend to prioritise when making a decision about taking part in the study?
- What questions do they typically ask?
- How long do mothers / partners have to make a decision about taking part in the study?
- Do they need any support or additional information when making this decision?
- Do you think there have been any barriers to recruiting parents into the study?
- How many people have declined to take part in the PLANES study and why?
- Concerning the written recruitment and participant information leaflets, have you had any questions or comments from parents concerning the information?
- Do you think written information is the best way to convey the trial or would a video or other methods be better?

*Discuss potential challenges to screening or systems that have been put in place to assist screening.*

**Section 4: Study conduct**

I have a few questions about your unit’s clinical management for these pregnancies

- How many women have you screened so far for SGA at the maternity assessment unit?
- Have you experienced any follow-up issues with women and birth partners after they’ve given consent to take part in the study? What about after they’ve given birth?
- Are there parts of the trial you have heard from women or birth partners that made them feel uneasy? *Explore more blood tests or more ultrasounds, deviating from standard NHS care*
- *How did you find entering data on RedCAP? Do you have a recommendation to better manage the data?*
- Have there been any issues following the study protocol or with sample storage and processing the samples?

**Section 5: Study Acceptability**

- Please have a look at the study flow chart. If its ok I would like to talk though each trial arm. Please let me know if you have any comments or concerns as I do in terms of how we should conduct the proposed clinical trial.
- (Go through flow chart one arm at a time)
- Do you think the PLANES feasibility trial design (refer to flow chart) is acceptable to conduct as a clinical trial?  *yes / no Explore: reasons and changes needed.*
- Do you have any concerns about the PLANES trial design?
- What, if any, barriers have you experienced in participating as a practitioner in this trial? *Explore practical or logistical challenges*
- What do you think would help facilitate a larger trial?

**Section 6: Training and Resources for Proposed PLANES RCT**

- Is there anything specific that you would suggest we include in the proposed PLANES trial site training package? If so, what would be helpful?
- Do you envision any potential barriers to training staff for the proposed PLANES trial?
- Are there any particular resources or other support that you would need to deliver the proposed PLANES trial?

**Section 7: Parent feedback**

- Overall, the feedback from PLANES participants has been positive, however; there have been some suggestions from parents on how to make their experiences better.
  - *Explore participant feedback about needing mental health support, continuity of care, how to support women whose choice as to place and mode of birth has changed*
- How feasible is it to include guidance or resources for parents who may need emotional support during their time in the trial? Especially for parents who have experienced baby loss before for SGA.
- A couple of mothers expressed they preferred to work with the same midwife and same consultant throughout their pregnancy rather than seeing different people. Is it possible to have a continuity of care pathway for parents who take part in the study?

**Section 8: Additional Comments & Closing Remarks**

- Is there anything else you would like to share that we have not already covered?

*Thank you for your time today. Explain next steps.*
