# Supplementary material for: Placental Growth Factor Led Management of the Small for Gestational Age Fetus: Randomised Controlled Feasibility Study
Source: BJOG. 2025 Dec 12;133(4):626–37. doi: 10.1111/1471-0528.70106 (PMC12884213; doi:10.1111/1471-0528.70106)
Supplement: Supplementary file 4 — Data S3: Topic Guide S2: Clinician interview and focus group topic guide. [file BJO-133-626-s004.docx]

**PLANES: Social Media Interview Topic Guide**


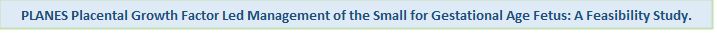


Please note: *Italic text indicates instructions for the researcher and will not be read to participant*

Introduction: My name is (researcher name) and I am a researcher from the University of Liverpool.  Many thanks for agreeing to help us with this study.

Before we begin the interview I need to check your consent for the study is that ok?

Do you consent for the interview and this consent discussion being audio recoded?

Do you consent to take part in an interview?

Do you understand that brief quotations from some interviews may be included in study reports.  Do you understand that nobody will be able to identify you in these reports?

*Reminder… if you find any questions difficult or you would not wish to answer them then please say and we can move on to the next question.  You can stop the interview at any time. Before we start do you have any questions?*

**I will start with some questions about you if that’s ok and then I will ask you about your experience of being invited to take part in the PLANES study**

**Section 1. BACKGROUND**

- Please could you tell me your date of birth?
- What is the first part of your postcode?
- Are you employed? If yes could you please describe your occupation?
- Could you tell me a little bit about when you first heard that your / your partner’s baby was small for gestational age?
- Could you tell me when you first heard about the PLANES study?  Could you tell me what was explained to you at that point in time?  (*Briefly explore clinical discussions that took place not study discussions.  Be empathetic and acknowledge this must have been a difficult time*)
- Explore how SGA was explained to the parents and by whom they first heard through midwife/doctor/ researcher
- Explore circumstances: *when did this occur?  Were you / your partner attending a check-up or scan?  Was this on a fetal medicine or maternity assessment unit?*
- How many weeks gestation were you / your partner at that point in time?
- How long ago was this?  *Date and work out current gestation / if given birth then how many weeks old.  Check on how child is now*

**Section 2. APPROACH TO CONSENT IN PLANES**

- We are in the process of developing a type of study called a clinical trial. Have you ever heard of a clinical trial before?
- Explain: *This is a type of medical research which provides information on the safety and effects of a drug, medical device, or intervention. They are used to find out the best way to treat patients in the future. Trials are carried out to test many different treatment plans for expecting parents.*
- **Based on the information provided in the PIS, ask participant to run through their understanding of what the PLANES trial is proposing. Talk through the PLANES PIS.**
- **Read social media PLANES aims and objectives**

Being small for gestational age (SGA) in the womb is a common condition affecting 1 in 10 of all pregnant women. If not managed with careful monitoring having a small baby may be related to a higher risk of stillbirth. At present there is no treatment available for small babies whilst still in the womb and current care on the NHS consists of careful monitoring with ultrasound and heart rate tracings (CTG) and early delivery, usually offered around 38 weeks. Early delivery requires interventions such as ‘starting off' (inducing) labour, when most of these babies are still healthy. When a small baby is delivered early, even from 36 weeks onwards, it can increase the risk of them having later health and developmental difficulties. Delaying delivery by even a few weeks could be of benefit to the baby’s development. We feel that if we have further reassurance of a baby’s health, such as how the placenta (afterbirth) is working, we can safely keep baby inside for longer.

Many studies using a blood test called the sFlt-1/PlGF ratio which measures indication levels in the blood, have shown that women with a normal test result are very unlikely to end up with a poor pregnancy outcome, such as stillbirth. It may be possible to use this blood test to give doctors and families reassurance that a baby’s placenta is working well and that doctors can safely leave baby inside the womb for longer, prolonging pregnancy and improving the health of babies. This will also allow doctors to keep pregnancies more normal and may prevent us needing to induce labour. The best way to find out if using this blood test to inform pregnancy management improves outcomes for babies is to conduct a clinical trial.

**Show or refer to PLANES flowchart and how trial participants are allocated into different arms. Explain randomisation**

**Section 3. PLACE IN CONTEXT OF PLANES TRIAL**

Imagine you were approached to take part in the PLANES trial after you received a growth scan indicating your baby was measuring small. The midwife explains the trial and asks if you would like to participate.

- What would be your initial thoughts about taking part in this trial?
- What are the initial benefits and risks of the study?
- Would you have any concerns about the PLANES trial, if so, what are they?
- Would you have any questions about the PLANES trial and what might these questions be?
- Looking at the information sheet and considering the flowchart, are there any parts of the study design that you think parents may find difficult to understand? *(Prompt: were there any parts of the information sheet that stood out to you in terms of influencing your decision to as to whether or not you would like to take part?)*
- Do you have any suggestions about how this discussion between healthcare providers and parents could be carried out to encourage parents to participate in the PLANES study?
- How much time would you need to consider the information before making a decision about the PLANES trial?

**Section 4. CONSENT DECISION MAKING**

- How long would it take you to think about whether you / your partner wanted to participate in the PLANES study?
- Would you discuss the study with your partner, friends or family to assist you in making a decision to take part? (Explore: *did their views influenced decision making?*)
- How long do you think people should be given to think about taking part in this study?
- In making the decision about participation in the PLANES study, what sort of things would go through your mind? (Prompt: *any concerns about blood tests?*)
- Explore: *any* ***perceived benefits*** *of being randomised to a) concealed blood test results b) revealed blood test* *results.  Would these perceived benefits impact your decision to take part in the study?*
- Explore: *any* ***concerns*** *about being randomised to a) concealed blood test results b) revealed blood test results.  Would you voice these concerns to the midwife / doctor?  Did these concerns impact your decision to take part in the study?*
- Would you / your partner have any concerns about the additional hospital visits that formed part of the study?  How important was this aspect in making a decision about the study?
- Would there be anything you could find particularly helpful in making up your mind about taking part in the PLANES study?  Was there anything you about the study’s information sheet you found unhelpful?
- How would you feel if you were randomized to a) concealed test results or b) revealed test results? Are there any concerns?
- What would be reason for saying no to the trial?
- Explore: *if this was before or after randomisation.  If after, explore concerns about allocation.*

**Section 5. TRIAL ACCEPTABILITY**

- Based off of the information provided and what we discussed, do you think this study is acceptable to be conducted as a full clinical trial?
- Explore: *response*
- Do you have any suggestions about how we could improve the consent process or any aspect of the study in the future?

*Thank the participant for their time and inform them how the results will be disseminated.  Send out a voucher and a copy of consent form if telephone interview.*
