# Supplementary material for: Placental Growth Factor Led Management of the Small for Gestational Age Fetus: Randomised Controlled Feasibility Study
Source: BJOG. 2025 Dec 12;133(4):626–37. doi: 10.1111/1471-0528.70106 (PMC12884213; doi:10.1111/1471-0528.70106)
Supplement: Supplementary file 5 — Table S1: Protocol amendments after the publication of the protocol. [file BJO-133-626-s005.docx]

| **Original Version** | **Original Date** | **New version** | **New date** | **Submitted to** | **Summary of Changes** |
| --- | --- | --- | --- | --- | --- |

| **VERSION 2.0** | **06-Jan-2022** | **VERSION 3.0** | **03-Nov-2022** | **Sponsor**  **REC**  **HRA** | - **Amendment to St Mary’s Hospital Laboratory contact (New contact - Alexandra Hasmi)** - **Amendment to Figure 1: PLANES study consort diagram** - **Amendment to participant management pathway:** - **Randomised Concealed**: **cCTG/USS as per local guideline, sFlt-1/PlGF Ratio and Research bloods at enrolment only** - **Randomised Revealed Normal Ratio: Research bloods at enrolment only, cCTG every 2 weeks** - **Randomised Revealed Abnormal Ratio: cCTG every week, no further sFlt-1/PlGF ratio or research bloods** - **Observational: Routine care, single sFlt-1/PlGF ratio at enrolment, offer delivery as per local clinical team** - **Inclusion of qualitative interviews for women (and partners) whose baby was small for gestational age in the womb within the last three years** - **Re-inclusion of section 11.5.4 Record Retention** |
| --- | --- | --- | --- | --- | --- |
