# Supplementary material for: Placental Growth Factor Led Management of the Small for Gestational Age Fetus: Randomised Controlled Feasibility Study
Source: BJOG. 2025 Dec 12;133(4):626–37. doi: 10.1111/1471-0528.70106 (PMC12884213; doi:10.1111/1471-0528.70106)
Supplement: Supplementary file 7 — Table S3: Reasons for timing of birth outside of protocol in those in the biomarker led, normal arm of the study. [file BJO-133-626-s003.docx]

| **Reason for birth <40^+0^ weeks’ gestation** | **n (percentage)** |
| --- | --- |
| Deviation from protocol | 9 (27.3%) |
| Growth concerns (static or reducing growth velocity) | 3 (9.1%) |
| Abnormal Dopplers or CTG concerns | 4 (12.1%) |
| Reduced fetal movements | 6 (18.2%) |
| Maternal conditions (preeclampsia, epilepsy, cholestasis) | 3 (9.1%) |
| Spontaneous labour or spontaneous rupture of membranes | 8 (24.2%) |
| **Total** | 33 |
