# Supplementary material for: Placental Growth Factor Led Management of the Small for Gestational Age Fetus: Randomised Controlled Feasibility Study
Source: BJOG. 2025 Dec 12;133(4):626–37. doi: 10.1111/1471-0528.70106 (PMC12884213; doi:10.1111/1471-0528.70106)
Supplement: Supplementary file 8 — Table S4: Health economics results. (A) Demographic characteristics of participants with complete and incomplete EQ‐5D data. (B) EQ‐5D completeness by randomisation arm. (C) Frequencies of EQ5D profiles at baseline and follow‐up. At time T1 (before delivery), the most commonly reported health state was that of ‘no problems’ in any of the EQ‐5D dimensions (11111), whilst in T2 (after delivery), the most commonly reported state was of some pain/discomfort, but no problems in other dimensions (11121). (D) Summary statistics for EQ‐5D index (utility) scores at times T1 (Enrolment) and T2 (Post‐delivery). EQ‐5D profiles were converted into index values (utility values) using the value set currently recommended by the National Institute for Health and Care Excellence in the UK1. On average, participant in the intervention arm had a higher EQ‐5D index (utility) scores than their counterparts in the standard care arm and observation arm. However, whilst utility scores in the intervention arm decreased markedly between T1 (enrollment) and T2 (post delivery), utility scores for the other arms remain at the same level or increased modestly. 1NICE. Position Statement on Use of the EQ‐5D‐5L Value Set for England. [file BJO-133-626-s001.docx]

**(A)**

| **Demographic characteristics** | **EQ-5D complete (n=49)** | **EQ-5D incomplete (n=29)** |
| --- | --- | --- |
| **Age** | 30.00 | 29.66 |
| **BMI** | 29.35 | 28.46 |
| **No. of previous pregnancies** | 2.37 | 1.90 |
| **Smoking status** |  |  |
| Never Smoked | 63.27% | 62.07% |
| Current Smoker | 18.37% | 17.24% |
| Stopped before pregnancy | 14.29% | 13.79% |
| Stopped in pregnancy | 4.08% | 6.90% |
| **No. of cigarettes smoked per day** | 0.98 | 1.48 |
| **Gestational age at enrolment (weeks)** | 33.86 | 33.66 |
| **Estimated Fetal Weight (grams)** | 1899.19 | 1893.08 |

**(B)**

| **Randomisation arm** | **EQ-5D complete (%*)** | **EQ-5D incomplete (%*)** |
| --- | --- | --- |
| Standard care (n=16) | 11 (68.75%) | 5 (31.25%) |
| Intervention (n=51) | 33 (64.71%) | 18 (35.30%) |
| Observation (n=11) | 5 (45.45%) | 6 (54.54%) |

**(C)**

|  | Baseline (T1) | |  | Follow-up (T2) | | |
| --- | --- | --- | --- | --- | --- | --- |
| EQ5D profile | Frequency | Percentage |  | EQ5D profile | Frequency | Percentage |
| 11111 | 19 | 32.20% |  | 11121 | 9 | 18.37% |
| 11121 | 10 | 16.95% |  | 22221 | 5 | 10.20% |
| 11112 | 4 | 6.78% |  | 11111 | 4 | 8.16% |
| 11122 | 3 | 5.08% |  | 21222 | 2 | 4.08% |
| 21221 | 2 | 3.39% |  | 21223 | 2 | 4.08% |
| 21222 | 2 | 3.39% |  | 21332 | 2 | 4.08% |

**(D)**

|  | **Intervention arm** | | **Standard care arm** | | **Observation arm** | |
| --- | --- | --- | --- | --- | --- | --- |
| **EQ-5D index scores** | **T1** | **T2** | **T1** | **T2** | **T1** | **T2** |
| Mean | 0.844 | 0.679 | 0.773 | 0.783 | 0.632 | 0.744 |
| SE | 0.022 | 0.043 | 0.071 | 0.039 | 0.111 | 0.057 |
| Lower 95% CI | 0.800 | 0.592 | 0.617 | 0.696 | 0.346 | 0.587 |
| Upper 95% CI | 0.888 | 0.766 | 0.928 | 0.871 | 0.918 | 0.902 |
| Min | 0.508 | -0.233 | 0.188 | 0.618 | 0.269 | 0.585 |
| Max | 0.985 | 0.985 | 0.985 | 0.985 | 0.985 | 0.871 |
